# Lessons learnt? The importance of metacognition and its implications for Cognitive Remediation in schizophrenia

**DOI:** 10.3389/fpsyg.2015.01259

**Published:** 2015-09-01

**Authors:** Matteo Cella, Clare Reeder, Til Wykes

**Affiliations:** Department of Psychology, Institute of Psychiatry, Psychology and Neuroscience, King's College LondonLondon, UK

**Keywords:** schizophrenia, cognition, metacognition, psychological therapy, learning, awareness, recovery

## Abstract

The cognitive problems experienced by people with schizophrenia not only impede recovery but also interfere with treatments designed to improve overall functioning. Hence there has been a proliferation of new therapies to treat cognitive problems with the hope that improvements will benefit future intervention and recovery outcomes. Cognitive remediation therapy (CR) that relies on intensive task practice can support basic cognitive functioning but there is little evidence on how these therapies lead to transfer to real life skills. However, there is increasing evidence that CR including elements of transfer training (e.g., strategy use and problem solving schemas) produce higher functional outcomes. It is hypothesized that these therapies achieve higher transfer by improving metacognition. People with schizophrenia have metacognitive problems; these include poor self-awareness and difficulties in planning for complex tasks. This paper reviews this evidence as well as research on why metacognition needs to be explicitly taught as part of cognitive treatments. The evidence is based on research on learning spanning from neuroscience to the field of education. Learning programmes, and CRT, may be able to achieve better outcomes if they explicitly teach metacognition including *metacognitive knowledge* (i.e., awareness of the cognitive requirements and approaches to tasks) and *metacognitive regulation* (i.e., cognitive control over the different task relevant cognitive requirements). These types of metacognition are essential for successful task performance, in particular, for controlling effort, accuracy and efficient strategy use. We consider metacognition vital for the transfer of therapeutic gains to everyday life tasks making it a therapy target that may yield greater gains compared to cognition alone for recovery interventions.

## Introduction

Across diagnoses the defining feature of mental ill health is impairment in the ability to function, which often translates into difficulties in attaining personal objectives or achieving expected goals. People with schizophrenia are often (but not always) at the most severe end of the functional disability spectrum and these difficulties, once established, tend to last a long time and affect all aspects of their life. Functioning difficulties are further limited by reduced normative developmental experiences, such as fewer or disrupted years in education, loss of friends or a lack of opportunity to make them, arising as a result of mental health problems. These may be particularly marked in people with schizophrenia as the disorder starts early and so there is less chance of learning or practicing skills important for future achievements. Society further limits opportunities through discrimination and stigma which prevent testing or practicing skills. Although these societal limitations are being addressed by campaigns (Evans-Lacko et al., 2013; Henderson and Thornicroft, 2013; Wykes, 2013) it is likely to take many years to reduce these effects.

There is consensus that cognitive difficulties in different domains including memory, attention, information processing speed and executive function play a relevant role in influencing functional difficulties and limiting recovery in people with a diagnosis of schizophrenia (Allott et al., 2011; Cella and Wykes, 2013; Miles et al., 2014). This prompted the development of therapies targeting cognition. Pharmacological therapies designed to target symptoms have a limited impact on cognitive difficulties (Keefe et al., 2007). Recently there has been an interest in developing medications to enhance cognition but most studies tend to report no boosting effects in uncontrolled trials (Freedman et al., 2008; Keefe et al., 2013). Psychological and behavioral interventions have more successfully been developed to fill this gap. Cognitive Remediation (CR) was designed to target cognitive problems with the broader aim of improving functioning. There is evidence that CR is beneficial but there is still a limited understanding of how the putative active therapy ingredients contribute to changes in functioning (Wykes et al., 2011; Cella et al., 2015). CR is designed to provide intensive practice in both basic and high level cognitive functions and the evidence suggests that supplementing cognitive task practice with strategy use can achieve higher returns in terms of functional gains (Wykes et al., 2011). This has prompted research into the mechanisms that may facilitate transfer of therapy gains into everyday life functional changes and can support recovery (Wykes et al., 2012). This paper will focus on the key role of metacognition in aiding transfer of therapy gains to everyday life.

## Unraveling metacognition

Metacognition has various definitions and applications across different fields. Flavell first used this term to define the cognitive process that relate to “thinking about thinking” (Flavell, 1979). Since its first definition, many authors have contextualized this concept to specific approaches and adapted and elaborated on its original meaning. The main developments have been in the sphere of pedagogy and the concept has driven much of the innovation in learning and teaching over the past 20 years (Education Endowment Foundation, 2013). In the domain of psychopathology the concept has received a high level of attention as problems in metacognition are thought to be implicated in a large number of higher level mental functions including self-reflection, introspection and behavior implementation. Problems in these functions are cardinal features of a number of severe mental health conditions including borderline personality disorder and psychosis (Bateman et al., 2007; Liotti and Gilbert, 2011). In people with schizophrenia the term metacognition is used by different proponents with diverse meanings and implications for outcomes and therapy. To avoid confusion it is useful to delineate briefly the different uses of the term.

### Metacognition in narrative

Lysaker and Dimaggio (2014) consider that problems in the sphere of metacognition affect the ability of people to make sense of their illness experience and compromise the integrity of their personal goals. Difficulties in these mental functions become evident when individuals are engaged in processes requiring an understanding of their own and other people's mental processes but also when this information is required to be mastered for social use. This has led these researchers to integrate their metacognitive approach as part of cognitive behavior therapy protocols and evaluate narrative coherence following therapy as a measure of metacognitive improvement (Lysaker et al., 2002; Wiffen and David, 2009).

### Metacognition and illness insight

A different approach is to consider metacognition as the cognitive function responsible for insight (David et al., 2012). A wealth of research suggests that the ability of people with schizophrenia to think about their symptoms (e.g., thinking about their delusion) is compromised (Koren et al., 2013; Nair et al., 2014). Limited insight and illness awareness have been associated with poor outcomes in people with psychosis (Frith, 2004) and changes in this function associated with clinical improvement (Corcoran and Frith, 2003). Despite a shared sense amongst clinicians that this may be an important domain to target there are no specific interventions for this domain.

### Metacognitive self- and cognitive-control

Self-related cognitive processes have been extensively linked to metacognition and considered important in influencing psychotic symptom development and maintenance. People with psychosis have negative beliefs about themselves and display unhelpful coping strategies toward their psychotic symptoms (Pickup and Frith, 2001). Coping strategies may be controlled by metacognitive beliefs and difficulties in this domain may influence illness outcomes. A study by Morrison and Wells (Morrison and Wells, 2003) reinforced this idea by suggesting that people who experience hallucinations, but do not develop schizophrenia, have higher levels of cognitive control compared to people with schizophrenia. This stresses the importance of metacognitive control and appraisal of psychotic experiences as a factor contributing to transition to schizophrenia and illness prognosis. This concept has been reformulated in a variety of ways, and is incorporated into different models of psychological therapies for psychosis (Tan, 2009; Ward et al., 2014).

Self-related concepts feature implicitly in another prominent theory of psychotic symptoms development. Frith (2004) proposed that psychotic symptoms result primarily from the inability to represent one's own and other people's mental states. This cognitive function is now widely referred to as theory of mind but can be seen as a metacognitive ability. According to Frith psychotic phenomena, such as thought insertions or delusions of control, are dependent on the inability to correctly represent intentions and actions or to exert monitoring and control over cognitive operations (Frith, 2004). Similarly, other proponents have elaborated on this idea and proposed that psychotic symptoms may result from problems in source monitoring (Keefe et al., 1999) through as the difficulty in distinguishing between the origins of self-generated and externally generated stimuli. Source monitoring can be seen as a metacognitive component providing agency information and facilitating the appraisal of events and life situations.

### Metacognition and thinking bias

It is well established that people with schizophrenia have a number of thinking biases which influence the development and maintenance of key psychotic symptoms such as delusions. These biases are targeted by psychological interventions such as Cognitive Behavioral Therapy for psychosis (CBTp) (Wykes et al., 2008) and specifically by Metacognitive Training (MCT) (see this issue [insert issue references]). Therapy focusses on improving awareness and mastery of the cognitive processes leading to erroneous conclusions (e.g., overconfidence).

### Metacognition and cognition

Most definitions described above consider metacognition as the function responsible for regulating thoughts, emotions and beliefs. An alternative, but not opposing, view characterizes metacognition as the process that regulates learning and information processing. This is not a new idea in psychology and has roots in Vygotsky's theories of learning potential but has been revisited more recently by Flavell (1979). Here the components of metacognition are: ***monitoring*** (evaluation of cognitive functioning), ***control or regulation*** (directing and evaluating cognitive and behavioral performance), and ***knowledge*** (understanding task difficulty and the resources required).

Awareness of cognitive problems can be thought of as a form of metacognitive knowledge that can effectively guide the deployment of cognitive resources to a specific task. This knowledge is essential for individuals to access the relevant resources required for maximal efficiency (Flavell, 1979). Individuals with a diagnosis of schizophrenia exhibit a mismatch between the subjective awareness and objective performance on cognitive tasks usually in the direction of under-estimating the problems (Cella et al., 2014). This mismatch indicates this lack of ***metacognitive knowledge***. Evidence of poor ***metacognitive regulation*** problems comes from the studies showing difficulties in executive functioning and in the control of decisions (Koren and Harvey, 2006; Koren et al., 2006). This concept coincides with executive function and is thought as the process that regulates and controls cognitive functions including working memory, attention, reasoning and information retrieval (Elliott, 2003). This process is often involved in tasks requiring the coordination of complex cognitive operations such as changing a plan in view of freshly gathered information, generating strategies, solving unexpected problems and organizing sequences of behaviors to accomplish a task.

It is possible to consider metacognitive ***knowledge*** and ***regulation*** as a hierarchy of mental processes referring to cognitive operations with some of these processes being more complex than others (Table 1). This framework may be useful in the context of therapy to identify competence levels at the beginning of an intervention and to consider progression milestones in mastering metacognitive skills.

**Table 1:** **Shows proficiency levels (with examples) of metacognitive knowledge and regulation**.

**Metacognition type and level**	**Proficiency level**	**Example**
*Knowledge 1*	The person is aware that cognitive operations are necessary for accomplishing everyday life tasks	Following a conversation is hard work and very confusing
*Knowledge 2*	The person has an understanding of the mental processes necessary to complete specific tasks	Following a conversation is hard because you have to pay attention to what the person is saying and remember the information
*Knowledge 3*	The person understands the impact of specific cognitive operations and associated difficulties on everyday life tasks and operations	I have problems in remembering people's name and because of that people sometime think that I'm rude
*Regulation 1*	The person has suboptimal adjustment to compensate for cognitive difficulties	I only pick up leaflets and small books because I'm not good at reading (e.g., avoidance)
*Regulation 2*	The person can anticipate some demands, shows limited degree of adaptation and planning	Studying in a quiet environment helps concentration but it is hard to retain information
*Regulation 3*	The person regularly uses strategies, adapts cognitive effort to task demands and can improve performance given practice and feed-back (e.g., learning from experience)	If I'm rested, I take notes and rehearse the material a couple of times I'm more likely to remember information. If there is too much to learn I can divide information in manageable chunks and take breaks

For the purpose of this paper we define metacognition as the process that regulates learning and information processing via metacognitive knowledge and regulation processes.

## Metacognition and functional outcomes in people with psychosis

Functional and recovery outcomes are poor in individuals with a diagnosis of schizophrenia in terms of work (Turner et al., 2015), their self-care and their relationships (Combs et al., 2011). The associations of poor functional performance with cognition are now well established but even though cognitive and social cognitive skills play a crucial role in influencing functional and treatment outcomes, it is not clear *how* they are related (Galderisi et al., 2014; Green et al., 2015). Studies conducted in the field of CR have highlighted the limited direct effect that enhancing cognition alone has on functioning suggesting that more complex mediating and moderating factors may be implicated in explaining the effects of the therapy (Wykes and Spaulding, 2011). More recently empirical evidence and theoretical accounts have emerged suggesting that metacognitive skills may be relevant in predicting functioning levels (Koren et al., 2006; Stratta et al., 2009; Hamm et al., 2012; Lysaker et al., 2013). This has prompted a number of studies specifically investigating the contribution that regulation and knowledge may have to functioning in people with schizophrenia. Stratta et al. (2009) first demonstrated that the association between metacognitive skills and functioning exceeds that of cognition with functioning when they showed that the relationship with cognition disappeared after testing a model including metacognition.

There is some confusion between executive function and metacognition. Metacognition requires some of the processes measured as executive functioning but is a wider concept involving decision making (see further explanatory examples in the discussion). Executive functioning, a concept akin to metacognitive regulation and monitoring, has been the focus of measurement which has demonstrated important links with clinical and functioning variables. In a recent study executive functioning was a significant predictor of duration of untreated psychosis (Fraguas et al., 2014) and individuals in remission were also found to have a higher performance on executive function tasks compared to non-remitted patients (Braw et al., 2012). These studies suggest links between illness symptoms and this cognitive domain. Executive function predicts supported and employment outcomes (Tan, 2009) and social functioning in people with schizophrenia receiving disability benefits (Tandberg et al., 2013). This suggests that aspects of metacognition captured by executive functioning measures are important to symptom and functional outcomes.

## Metacognition, learning and skills transfer

Given that people with schizophrenia have poor recovery outcomes, it is surprising that little research has concentrated on how gains made in therapy through CR or other therapies transfers into functional gains. Unlike in mental health, the field of education has stressed the importance of promoting transferable skills and evidence now exists for the mechanisms that may facilitate their acquisition and usage.

Methods such as problem based learning (PBL) allow learners to acquire knowledge by exposure to problems. Learners seek information that would reduce their uncertainty and self-guide their learning on the basis of problem demands. This method is often claimed to be the rationale for apprenticeships and it has been applied across disciplines from vocational training to medical education (e.g., Imanieh et al., 2014). Research in this area has also demonstrated the importance of the learning environment for generalization and transfer. Knowledge is learnt only as part of a unique context and it is less likely to generalize if the learning and the everyday life application contexts are different. This consideration stresses the importance of focussing on maximizing the opportunity for learners to acquire and maintain schemas that can be used in different situations. Providing practice in every possible environment where the schema may be needed is, however, impossible so the focus has shifted to which learning strategies may facilitate this transfer process. Abstract explanations can supplement practice (Anderson et al., 1996) but more recently the research focus has been on cognitive control and how the allocation of cognitive resources may influence learning, transfer and usage of learnt material in everyday life (Tullis and Benjamin, 2011).

These processes are generally referred to as metacognitive. Education reviews suggest that metacognitive skills can guide strategic learning by explicitly teaching strategies and knowledge use, and ensuring that learners use monitoring processes to implement and review their performance (Education Endowment Foundation, 2013). The effect of adopting this metacognitive approach to facilitate learning has tangible effects. In learning to read this method resulted in improvement of about 8 months of reading age suggesting this approach not only as effective but also cost effective (Dignath et al., 2008; Education Endowment Foundation, 2013). More recently, the connection between an individual's metacognition and learning and especially how it can be boosted has been investigated. Effective monitoring (part of metacognitive regulation) is essential for the self-management of learning (e.g., Anderson et al., 1996; Tullis and Benjamin, 2011) and improved monitoring accuracy increases the effective allocation of study time between different items as well as overall recall performance (e.g., Thiede et al., 2003). Recent reports also suggest changing metacognition by boosting awareness, e.g., “being aware of one's strengths and weaknesses as a learner, developing self-assessment skills, and being able to set and monitor goals,” and a repertoire of strategies to choose from during learning are vital to improve learning (Education Endowment Foundation, 2013). So there is evidence that targeting metacognition can improve learning and recall and improves generalization to other tasks. This is achieved through explicit teaching of strategies for learning and generalization to other situations.

## Cognitive remediation and metacognition

With learning being the vehicle of change in most psychological therapies it is perhaps surprising that metacognition has only recently started to feature in CR descriptions. Even then there is still little emphasis on how it builds functional benefits. For CR the primary target is cognition as changes are thought to exert an effect on functioning, however, there is limited knowledge on how the transfer from improved cognition to improved functioning may work. Here we will describe how we think metacognition should be used as part of CR to maximize transfer and functional gains.

**Figure 1:**
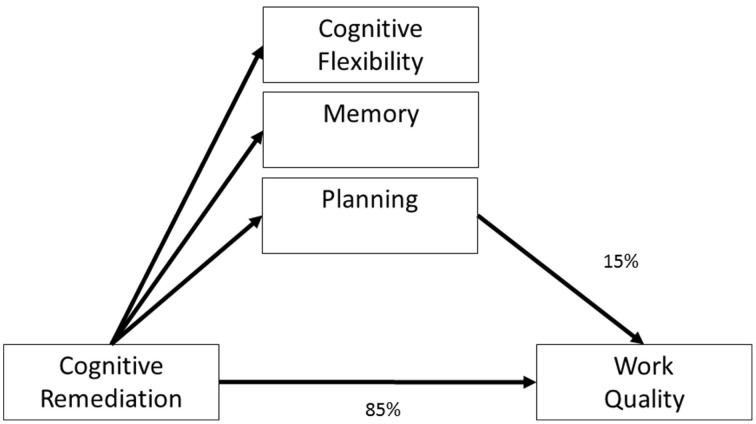
**A model of how cognitive remediation influences functional outcomes**. Adapted from Wykes et al. (2012).

**Figure 2:**
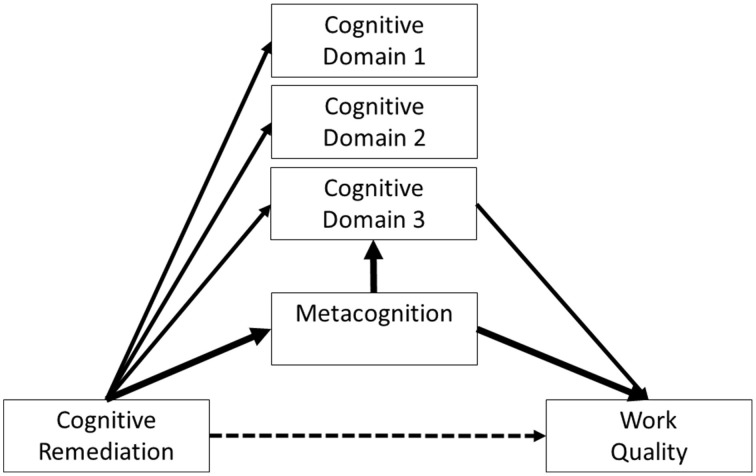
**A model of how metacognition can influences functional outcomes**.

The potential importance of metacognition in CR programmes comes from the evidence that everyday tasks and even the costs of care (a proxy for the level of functional support needed) are more likely to improve following improvement on executive tasks (i.e., changes in metacognitive regulation and executive functions) (Reeder et al., 2004, 2014; Eack et al., 2010a; Penadés et al., 2010; Wykes et al., 2012). Wykes et al. (2012) investigated the “how” of cognitive change impact on functional changes in a study of the effects of improved cognitive performance on work outcomes. The model tested is shown in Figure 1. However, mediation models of improving functioning suggest that the variance accounted for by improved cognitive performance alone is only 15%, leaving as much as 85% of variance unexplained. Notwithstanding the measurement problems in cognitive outcomes following remediation, we contend that some of this unexplained variance relates to skills learnt directly within CR that are immediately transferrable to everyday tasks and social relationships. Figure 2 provides this framework. Here CR changes metacognition and the cognitive tasks but it is mainly metacognition (indicated by the width of the line) which drives the effects on functional outcome. Performance on cognitive tasks may also improve not just through task practice but through improvements in metacognition. This new model suggests that cognitive task improvement is not a mediator but is a third variable affected by the mediator (metacognition) and that therapy should target metacognition.

So how might this inform a newer model for cognitive remediation? We can use some evidence already gleaned on types of programmes. Broadly speaking CR implementation methods can be differentiated into two “schools.” The first, the drill and practice approach, proposes that cognitive improvement can be obtained primarily by frequent and intensive task practice tailored to the individual's ability. The second school adopts a strategy approach, which suggests that intensive practice should be supplemented by explicit training of strategies and approaches to the tasks (Cella et al., 2012). Both these types of programme have shown to have an impact on neural plasticity and can alter functional and structural brain parameters including network efficiency and gray matter loss (Eack et al., 2010b; Subramaniam et al., 2012; Penadés et al., 2013; Pu et al., 2014). However, in meta-analyses the strategy based programs have greater and significant effects on transfer to functional outcomes, particularly in the context of opportunities for functional gains e.g., within rehabilitation programmes (Wykes et al., 2011).

Strategy use and the explicit attempt to increase knowledge about the individual's cognitive resources are metacognitive competencies (i.e., *metacognitive knowledge*). This is often explained to patients as the knowledge they have about their thinking in general including what they think they can do best (i.e., cognitive strengths) and what they have difficulties with. Metacognition is also applied in the context of CR as a process of recognizing the needs of a task and implementing the appropriate strategies and resources. This is referred to as *metacognitive regulation* which is generally divided into three sub-processes—planning, monitoring and evaluation. In the planning stage individuals bring to mind all the relevant information to complete a task and organize the stages into the necessary sequence for the task. In the monitoring phase the individual monitors actions as they are executed and adapts them if needed. In the evaluation stage the individual reconsiders the cognitive operations and evaluates their usefulness for similar future tasks. This process can be introduced to patients using the acronym PriME (i.e., Planning, Monitoring and Evaluation) and systematically applied to tasks during training. In the planning phase patients are encouraged to make plans formally before acting, to forecast possible difficulties in order to prevent problems. This includes non-cognitive factors such as, social anxiety, boredom and feeling low in mood. In the monitoring stage patients are encouraged to check the execution of the plan including making an assessment of the proficiency of the strategies used and flexibly adapt them to the situation. In the evaluation phase, patients are encouraged to assess and review their performance and consider what went well and what could have been done differently. The training supports the integration of this information in future planning.

## Applying metacognition to cognitive remediation

**Figure 3:**
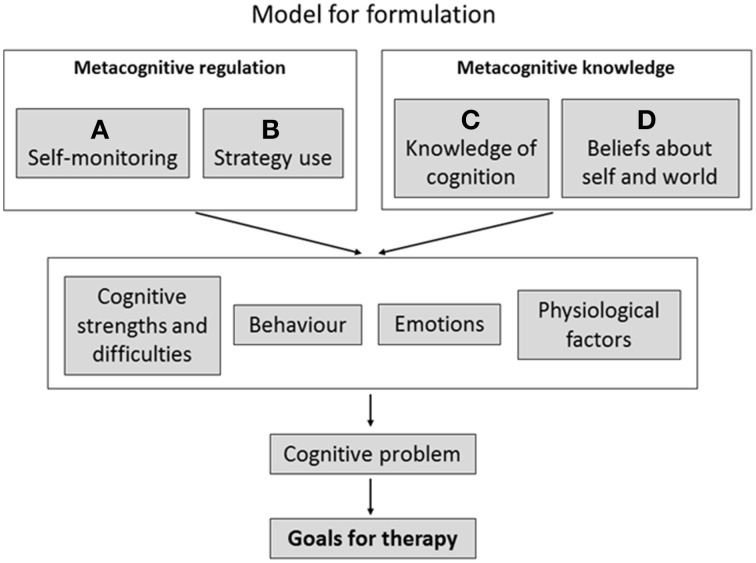
**Shows a formulation model using metacognition in the context of CR**.

The model adopted by Wykes and Reeder (2005; Wykes et al., 2007) integrated metacognition as part of CR and is shown in Figure 3. Here personal recovery-based goals are set with the client and these goals are ones that can be related to cognitive problems. We call these *Cog-SMART goals* (i.e., Cognition related- Specific- Measurable- Achievable- Relevant- Time specific) which are always embedded in real-world activities. These goals are usually formulated as a series of stages to demonstrate that the highly valued end goal is achievable and directly linked to the smaller goals set in therapy. For instance, a first step goal might be “improving my attention so I rely on fewer prompts in a session.” This may lead to a second step goal—“I will be able to describe what I am reading to someone”—and this in turn can lead to a third step goal—“I can communicate my needs and be understood.” Success in these milestones may also lead to the development of other personal goals, e.g., enroll in a carpentry course or go back to college, depending on the stage and competencies of the client. Goal development may depend on the client's competencies in metacognitive regulation and knowledge which can be extracted from metacognitive assessments or based on discussions with clients or observations of their behavior in cognitive assessments. Setting goals also requires a discussion of other factors that may affect cognitive outcomes e.g., when anxious you may not perform as well.

CR programmes can then aim to increase awareness of cognitive strengths and weakness that can be overcome using strategies developed in therapy. For instance, helping the individual become aware of mnemonic strategies that can aid memory and the situations where these strategies are likely to be required. This type of remediation emphasizes the explicit teaching of strategies and the prompting of awareness. This can be partially achieved through automatic mechanisms embedded in CR software. An example is the CIRCuiTS CR programme which contains not only tasks with a focus on cognitive skills but also prompt individuals to select and use strategies (Reeder et al., 2015). The software has basic cognitive tasks and exercises that closely resemble valued functional activities such as writing your cv, looking for a job, cooking a recipe, going shopping etc. In order to encourage metacognitive processing before each task it is essential to select one or more strategies and to predict how difficult the task might be and how long it might take. Following the task the client receives feedback which can aid monitoring. Task performance is tailored to 80% success to keep engagement and efficacy high. Once a task is completed the client is asked to rate how difficult they had found the task and how helpful the strategy had been. Building strategies in this programme is linked to improved functioning and cognitive improvements (Wykes and Cella, 2015).

## Discussion

### The optimism of a metacognitive approach

For individuals with a diagnosis of schizophrenia whose cognition is often poor, adopting a metacognitive approach has promise. Research in education suggests that this is an approach that is particularly beneficial for low achieving students or older people where larger improvements have been noticed (Education Endowment Foundation, 2013). This means that it may be particularly suited to individuals with a diagnosis of schizophrenia who have failed to benefit from usual rehabilitation or recovery programmes that provide direct on-the-job teaching. This notion is supported by recent research demonstrating the significant improvement in employment provided by CR in addition to work rehabilitation, but only in those who were already functioning at a lower level (Bell et al., 2014). The effect of CR in the higher functioning group was negligible. It may be that the metacognitive abilities of individuals in the higher functioning group were intact and only an opportunity for practice in a work setting and support to overcome stigma and discrimination in the workplace is required.

Metacognition also gives a structured approach to learning which has been absent for some time in therapy development. This learning approach allows the therapist to lead the client through the therapy with a clearer idea of what learning processes need to be in place. Many CR programmes already provide metacognitive input. For example any programme that mentions explicit teaching of strategy and help with reflection on task performance is essentially aiding metacognition. However, in many programmes this aspect has not yet been formalized as an “active therapy component” and we think this does not recognize the value of an important therapy ingredient.

### Where now with metacognition research?

We require more evidence on the role of metacognition in the context of all therapies but particularly in CR. The recognition of its importance needs to fuel assessment as part of therapy in order to bolster those aspects which are problematic. For instance, if a client had a store of metacognitive knowledge including effective strategies but little awareness of where and when they might be used then their therapy programme should emphasize metacognitive regulation.

We also have limited information on what may negatively influence metacognition. We know that in stressful situations our cognitive abilities are affected. If these situations do affect metacognition generally then encouraging alternative responses through methods other than cognitive remediation would be appropriate, including increasing self-esteem or targeted therapy for auditory hallucinations. However, the evidence on these potential therapy avenues is not clear and there is no specific recommendation as to how different interventions gains may contribute to global outcomes.

Evidence on self-awareness may throw some light on how sub-domains of metacognition, including metacognitive knowledge, may be changed by affective components. In one of our recent studies we demonstrated that when self-esteem levels were controlled, cognitive performance evaluations were more in keeping with the corresponding objective neuropsychological assessments (Cella et al., 2014). Self-esteem also affects strategy use in people with schizophrenia within a CR programme. Those with higher self-esteem used fewer strategies even though more strategy use increases the effects of therapy (Wykes and Cella, 2015). Here self-esteem may dampen the effects of CR and may be a factor important for therapists to monitor as a potential barrier to therapy-related improvements.

Currently, we do not know which tasks best facilitate the active use of metacognitive competencies. This information is vital if we are to improve the efficiency of therapy and laboratory task development can aid this selection. We also need to know how individual characteristics, e.g., poor cognitive reserve, can inhibit task efficiency. Studies of moderators and mediators of the treatment effects will contribute to clarifying the role of intensive training and strategy use but also to characterize these active CR building blocks by comparing different therapies.

The boundaries between cognition and metacognition are not distinct. Some domains of executive function, such as monitoring, overlap with metacognition and this makes the measurement of metacognition and cognition difficult to separate. CR programs that target executive function do seem to achieve higher functional gains but it is unclear if these gains are separate from or included in metacognition gains. In Figure 2 we suggest that metacognition may partially exert its influence on functioning via executive function but we propose that there may be also a direct link related to higher metacognitive competencies not captured by executive functions alone. For instance two individuals experiencing low mood may be equally able to make a good plan in relation to organizing a visit to a friend. However, only one may perceive the low mood as a limitation to the implementation of the plan and realize that it may not be a good day to travel while the second person may try and fail. Both these people may have similar scores on a planning assessment but may have different levels of metacognition.

## Conclusion

It has been 10 years since we first suggested the importance of metacognition (Wykes and Reeder, 2005) in the context of CR and since then this concept has become increasingly used. However, although this provides structure for learning and is supported by a long research programme in education, the term is being used too loosely. Therapy developers need not only to refer to “thinking about thinking” but to specify the concept in a way which allows its measurement. The ability of metacognition to be helpful is totally dependent on our ability to replicate the findings of others and to be less circular in our outcome assessment. To move forward the field needs better operationalization of the term, more rigorous measurement and testing in the context of interventions.

### Conflict of interest statement

The authors declare that the research was conducted in the absence of any commercial or financial relationships that could be construed as a potential conflict of interest.
